# Enhancing reaction-based de novo design using a multi-label reaction class recommender

**DOI:** 10.1007/s10822-020-00300-6

**Published:** 2020-02-28

**Authors:** Gian Marco Ghiandoni, Michael J. Bodkin, Beining Chen, Dimitar Hristozov, James E. A. Wallace, James Webster, Valerie J. Gillet

**Affiliations:** 1 Information School, University of Sheffield, Regent Court, 211 Portobello, Sheffield, S1 4DP UK; 2 Evotec (U.K.) Ltd, 114 Innovation Drive, Milton Park, Abingdon, OX14 4RZ UK; 3 Chemistry Department, University of Sheffield, Dainton Building, Brook Hill, Sheffield, S3 7HF UK

**Keywords:** De novo design, Reaction class recommender, Multi-label classification, Reaction vector

## Abstract

Reaction-based de novo design refers to the in-silico generation of novel chemical structures by combining reagents using structural transformations derived from known reactions. The driver for using reaction-based transformations is to increase the likelihood of the designed molecules being synthetically accessible. We have previously described a reaction-based de novo design method based on reaction vectors which are transformation rules that are encoded automatically from reaction databases. A limitation of reaction vectors is that they account for structural changes that occur at the core of a reaction only, and they do not consider the presence of competing functionalities that can compromise the reaction outcome. Here, we present the development of a Reaction Class Recommender to enhance the reaction vector framework. The recommender is intended to be used as a filter on the reaction vectors that are applied during de novo design to reduce the combinatorial explosion of in-silico molecules produced while limiting the generated structures to those which are most likely to be synthesisable. The recommender has been validated using an external data set extracted from the recent medicinal chemistry literature and in two simulated de novo design experiments. Results suggest that the use of the recommender drastically reduces the number of solutions explored by the algorithm while preserving the chance of finding relevant solutions and increasing the global synthetic accessibility of the designed molecules.

## Introduction

The aim of de novo design is to generate molecules in-silico to fit a set of design constraints [1]. The first methods for de novo design were described in the late 1980′s, however, despite approximately 30 years of research it remains a very challenging problem. The difficulties arise from the astronomically large number of compounds that could potentially exist and the complexities associated with the accurate scoring of potential solutions to drive the search towards desirable compounds [2]. Early approaches to de novo design were largely agnostic of chemical synthesis. Molecules were constructed either atom-by-atom or fragment-by-fragment using rules of chemical valency, with the result that the designed compounds were often unattractive to medicinal chemists. This lack of synthetic accessibility was a significant factor that limited the uptake of the early methods.

A significant advance in de novo design methods was the introduction of reaction-based methods in which the issue of synthetic accessibility is addressed directly [3, 4]. In reaction-based de novo design, reaction “rules” are used to transform a starting material into a product molecule. The rules are derived from commonly used reactions in medicinal chemistry, thereby increasing the chance that the in-silico molecules can be synthesised. The first generation of these approaches was based on hard-coded sets of reactions.

A more recent development in de novo design is the use of AI-based techniques which mainly rely on the use of generative and predictive models obtained using deep learning techniques [5]. For example, variational autoencoders have been used to map chemical structures represented as SMILES strings into a continuous latent space [6, 7]. New structures are generated by searching for optimal solutions in the latent space and then decoding these back to SMILES strings. Recurrent neural networks (RNNs) have also been applied to de novo design [8, 9]. The RNNs are first trained on large numbers of existing SMILES in order to learn the underlying probability distribution of the SMILES syntax, they are then used to generate new SMILES that are not present in the training data. Although promising, the AI approaches do not currently account for synthetic accessibility directly. It can be argued, however, that synthetic accessibility is implicit in the methods since the training data consists of real compounds thereby presenting a bias towards synthesisable molecules.

We have developed a reaction-based de novo design approach which, rather than being based on hard-coded reaction rules, is based on transformation rules which can be derived automatically from large sets of reactions [10, 11]. Our motivation has been to develop a tool that is able to exploit the wealth of information on chemical reactions that is available in publicly available data sets, commercial databases and in-house electronic lab notebooks (ELNs). The knowledge-base of transformations is separated from the structure generator so that the de novo design tool can be easily tailored, for example, to use reactions that are frequently encountered in medicinal chemistry, or to use more innovative, less well establish reactions, such as those used within academic settings. The transformation rules are encoded as reaction vectors which account for the structural changes that take place in converting one or more starting material(s) into one or more product(s). A structure generation algorithm has been developed whereby a reaction vector can be applied to an unseen starting material and, assuming the required elements of the reaction centre are present, be used to generate an in-silico product molecule. We have demonstrated a high degree of success in reproducing the known product(s) of a reaction based on known reactants and their associated reaction vectors [12]. In a recent publication we describe the generation of reaction vectors from the 1.8 million reactions extracted from the US patent literature in the development of a data-driven reaction classification method called SHREC [13]. SHREC takes a reaction vector as input and outputs a reaction class.

Although effective at reproducing known reactions, a limitation of the reaction vector approach to de novo design is that it does not take account of functionality outside of the immediate vicinity of the reaction centre. For example, the presence of a deactivating group beyond the reaction centre is ignored by the structure generation tool so that a molecule may be generated which, although consistent with a reaction vector, may not be synthetically accessible. Here we describe the development of a machine learning approach that aims to associate recommended reaction classes with a given input molecule. We have named our approach a Reaction Class Recommender. The recommender is trained using starting materials extracted from a large collection of reactions which are described using molecular features (e.g. fingerprints) and labelled by reaction class. The use of whole molecule descriptors allows the wider environment of a reaction to be encoded in the recommender. Starting materials described by the same sets of features (i.e., identical fingerprints) are grouped to form a single entry associated with multiple reaction classes. The recommender is then trained on sets of molecular features associated with multiple reaction classes, and, given a starting material represented by the same molecular features, it will output a list of recommended reaction classes. Training the recommender is, therefore, configured as a multi-label classification problem [14]. Multi-label classification approaches have previously been used to predict the activity profiles of small molecules against a panel of protein targets [15–19], drug side-effects [20], and to identify possible plant sources for natural products [21].

The reaction classes output by the recommender can be used in reaction-based de novo design to limit the reactions that are applied to those that are more likely to work in reality. For example, the recommender can act as a filter with only those reaction vectors that are within the recommended reaction classes being applied. In the case of fully automated de novo design, the recommended reaction classes can be used to automatically filter the reaction vectors which are applied to a given input molecule with the aim of reducing the chemical space that is enumerated. In augmented de novo design, the recommended reaction classes can be presented to the user to provide greater control over the design process.

The manuscript is organised as follows. We first present an overview of the approach before describing the methods in detail. The experimental section presents the results of an extensive set of parameterisation experiments aimed at determining the best performing Reaction Class Recommenders which are then evaluated in two different de novo design settings.

## Methods

### Overview

**Fig. 1:**
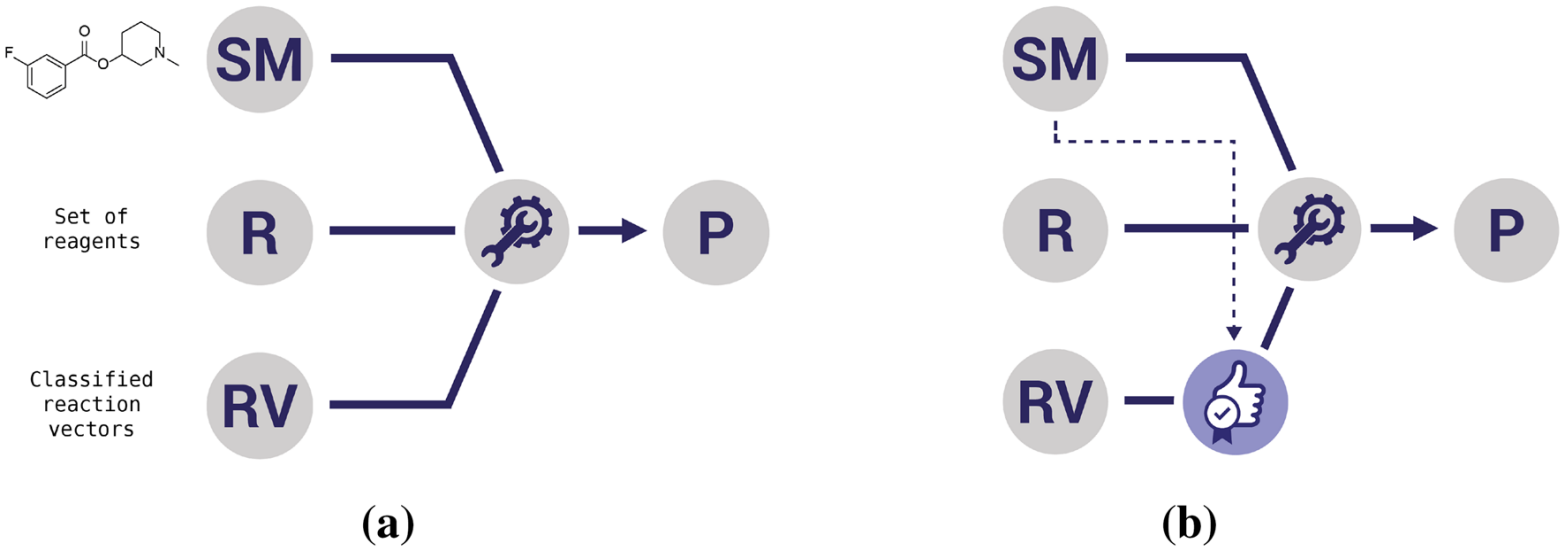
An overview of the use of the Reaction Class Recommender in de novo design. **a** Shows one iteration of the de novo design workflow consisting of one or more starting materials, a set of reagents and a set of reaction vectors. The reaction vectors are scanned for each starting material. Applicable reaction vectors are those for which the negative atom pairs are wholly present in the starting material or which are present in the starting material and a reagent. In **b** the Reaction Class Recommender is used to obtain a list of recommended reaction classes based on the characteristics of the starting material and only those reaction vectors in the recommended classes are considered further

The Reaction Class Recommender is designed to identify a set of recommended reaction classes for a given starting material in order to restrict the generated molecules to those that are most plausible for synthesis. Its intended use is shown in Fig. 1. The existing de novo design workflow [10, 11] is shown on the left and typically involves three sets of inputs: one or more starting materials (SM); a set of reagents (R); and a set of reaction vectors (RV). Reaction vectors are based on a combination of atom pair 2 and atom pair 3 descriptors (AP2 + AP3) [22] and are calculated from balanced reactions by first summing the descriptors for the products and the reactants, respectively, and then subtracting the reactant descriptors from the product descriptors. A reaction vector consists of negative atom pairs which represent those lost from the reactant(s) and positive atom pairs which represent those gained in the product(s). For each starting material, the set of reaction vectors is scanned and all those that are applicable are identified. A reaction vector is applicable if all of the negative atom pairs are present in either the starting material itself or in the starting material combined with a reagent. The applicable reaction vectors and optional reagents then form the inputs to the structure generator which attempts to generate a product molecule. The image on the right illustrates the use of the Reaction Class Recommender. A list of recommended reaction classes is generated for each starting material. The reaction vectors are then filtered and only those that are in the recommended reaction classes are taken forward for the applicability test and subsequent structure generation, thus limiting the products that are generated.

The Reaction Class Recommender is trained on a set of classified reactions. Each starting material is extracted, labelled according to the reaction class, and then represented by a set of whole molecule descriptors. Whole molecule descriptors are used since the aim is to include information beyond the immediate reaction centre and encode elements of the wider structural environment in which reactions occur. The de novo design will then be focused on typical starting material-reaction class combinations seen in historical data. Depending on the level of generalisation of the descriptors used to represent the starting materials, this process will result in duplicates whereby different starting materials are grouped according to identical descriptors. In this case, the entries are combined and their reaction labels aggregated. To take a highly contrived example, a molecule represented solely by an aldehyde group could potentially react with either a Grignard reagent or an ammonia derivative to form a C–C or a C–N bond, respectively. Assuming the training data contains examples of both reactions, the molecules (or more precisely the descriptor sets derived from the molecules) will be merged to a single input which is associated with two reaction class labels.

**Fig. 2:**
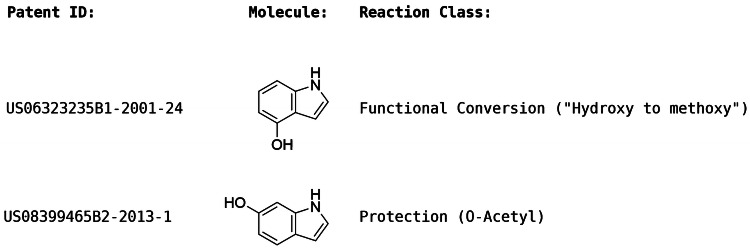
Examples of two molecules which are represented by identical functional groups consisting of the amine (NH) and hydroxyl (OH) groups only. They would be merged to a single entry in the training data which is associated with the two reaction classes shown

Another hypothetical example is reported in Fig. 2 to demonstrate the aim of the recommender. Two starting materials extracted from the US Patent Database (USPD) are shown together with their reaction class labels. Assume that the starting materials are represented by functional group descriptors, and that each starting material is represented by the presence of the amine (NH) and hydroxyl (OH) groups only. Given the identical descriptors, the two starting materials would be merged to a single entry with two reaction class labels; Functional Conversion and Protection. When these two functional groups occur within the same molecule they can compete for many reactions such as coupling and condensation reactions, and a chemist would be required to block one of them before proceeding with a reaction that could otherwise occur at multiple reaction centres. The reaction vectors alone are agnostic of the competing nature of the functional groups and may therefore result in in-silico products that are unlikely in reality. The recommender, however, will not suggest coupling or condensation reactions for a molecule that contains both these functional groups if there is an absence of such examples in the training data.

**Fig. 3:**
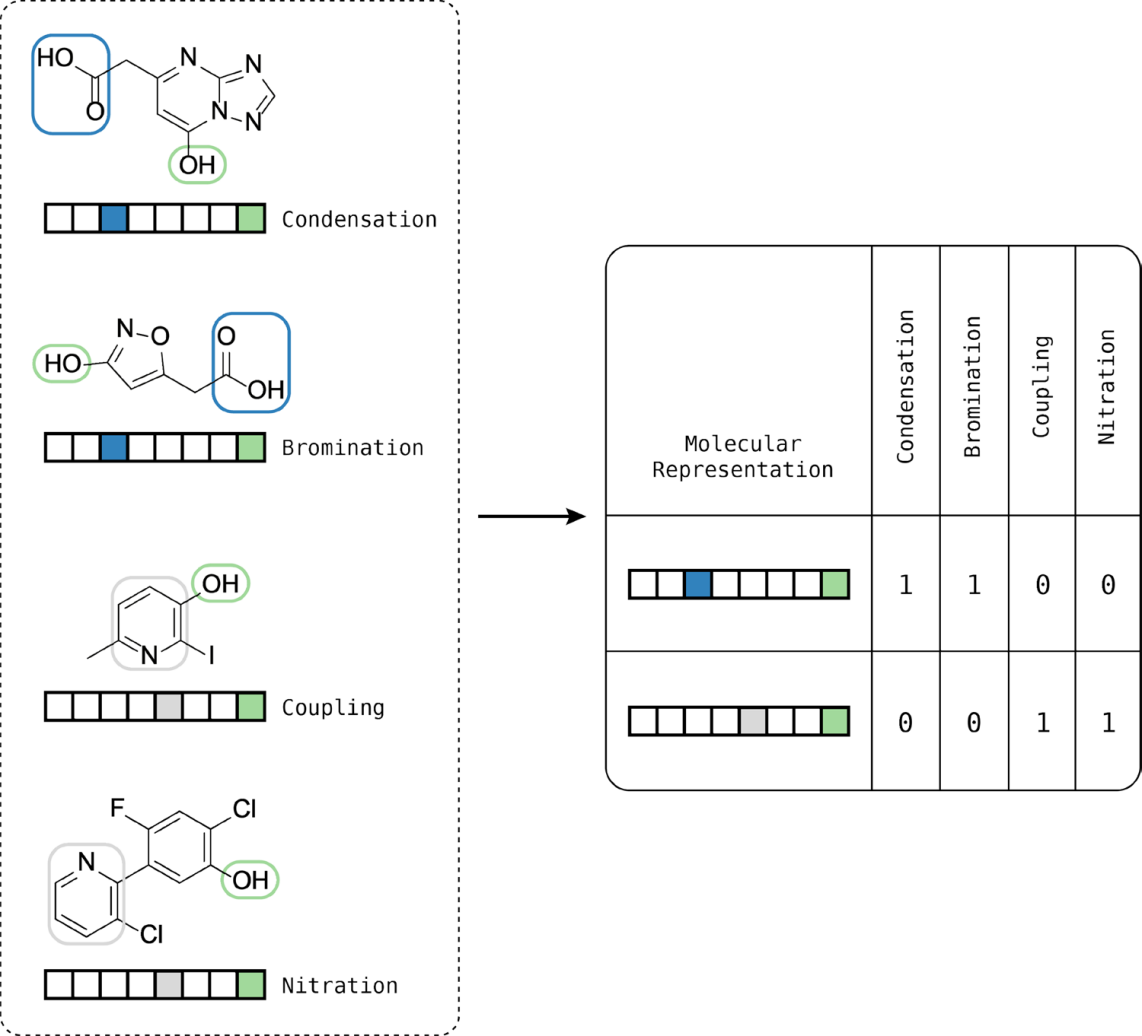
Starting materials are extracted from a set of classified reactions along with their associated reaction class label. The starting materials are characterised by whole molecule descriptors (represented as vectors) and duplicate descriptors are merged into a single entry by appending the appropriate reaction class labels. Thus, each entry in the training set represents one or more starting materials and a multi-label classification represented as a binary vector

The process for generating the training data for model building from a set of annotated starting materials is shown in Fig. 3. The process starts with a set of classified reactions. We use the hierarchical reaction classification system SHREC [13] which is compatible with the reaction vector approach and is summarised briefly below.

SHREC is distributed across four hierarchical levels ranging from general reaction categories to increasingly more specific subclasses. The hierarchy allows the most specific reaction classes to be merged into more generic categories (e.g., “C–C Bond Formation (Condensation)” and “C–C Bond Formation (Coupling)” can be merged into “C–C Bond Formation” by moving up a level in the hierarchy, and vice versa. The first (highest) level in the hierarchy describes a reaction according to some basic chemistry definitions (e.g., C–C bond formation, Functional Conversion, Protection, etc.); the second level describes the type of the transformation (e.g., Coupling), or in some cases, a specific substrate involved in the reaction (e.g., Alcohol to alkene). The third and fourth levels contain additional information on the substrates/products (e.g., Isocyanate + amine), reaction inventors (e.g., Suzuki) or functionalities (e.g., Bromo). The different levels in the hierarchy are shown by the use of parentheses. Examples are given in Table 1 for C–C bond formation reactions. The reaction classifier was trained on data extracted from the USPD which was originally annotated with NameRxn classes, and a table showing the mapping of the original NameRxn labels to the four-level SHREC is shown in the Supporting Information of Ghiandoni et al. [13]. Note that the four-level hierarchical labelling in SHREC is not exhaustive in terms of nomenclature due to its bias towards the USPD and NameRxn.

**Table 1:** Mapping of NameRxn labels to the SHREC for a set of C–C bond formation reactions

	SHREC			
NameRxn class	Level-1	Level-2	Level-3	Level-4
Bromo Heck reaction	C–C Bond Formation	Coupling	Heck	Bromo
Negishi coupling	C–C Bond Formation	Coupling	Negishi	–
Chloro Stille reaction	C–C Bond Formation	Coupling	Stille	Chloro
Iodo Sonogashira coupling	C–C Bond Formation	Coupling	Sonogashira	Iodo
Iodo Suzuki coupling	C–C Bond Formation	Coupling	Suzuki	Iodo

### Data

**Fig. 4:**
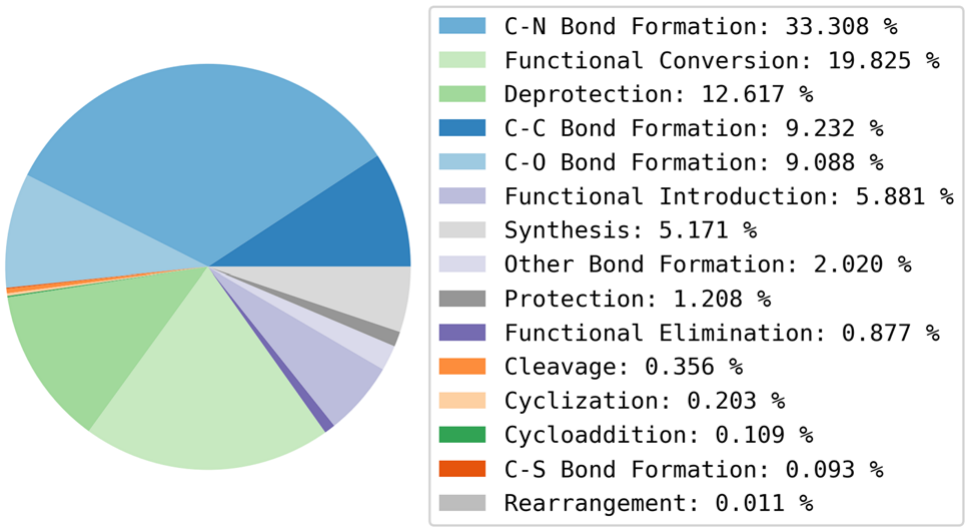
The composition of reaction classes in the clean USPD Grants subset using the highest level of the classification (level-1)

The training data is derived from USPD Grants 1976-2016 data set [23]. The original data set consists of approximately 1.8 million reactions of which 1.1 million are annotated with classification labels generated using the NameRxn classification tool. The classified reactions were extracted and the NameRxn labels translated to SHREC labels. A total of 735 NameRxn reaction classes were present in the data, however, the Reaction Classification model was trained on 336 of these only (a threshold of at least 30 examples per reaction class was used in that work and the removal of a large number of classes reflects the highly skewed nature of the USPD data). Reactions in classes that fall outside of the scope of the SHREC classifier were removed (approximately 5%) from the data set used to train the recommender, see Table 2. The composition of reaction classes in the USPD Grants subset is shown in Figure 4 for the highest level of SHREC (level-1).

**Table 2:** Training data

Data set	Number of entries	Number of classes	Median number of examples per class
USPD Grants NameRxn	1,114,953 reactions	735*	160
USPD Grants SHREC	1,056,836 starting materials	336**	799.5
Level-3 subset	430,543 starting materials	319	342
Level-2 subset	424,138 starting materials	259	290

*Reaction classes determined by the NextMove NameRxn algorithm

**Reaction classes determined using SHREC. There are fewer distinct reaction classes at Level-2 due to the duplicate removal stage. If a given starting material is associated with the reactions “C–C Bond Formation (Coupling) (Suzuki)” and “C–C Bond Formation (Coupling) (Heck)”, in the level-3 setup the entry will appear twice, while using level-2 (“C–C Bond Formation (Coupling)”), one of the two entries will be filtered out as a duplicate

**Fig. 5:**
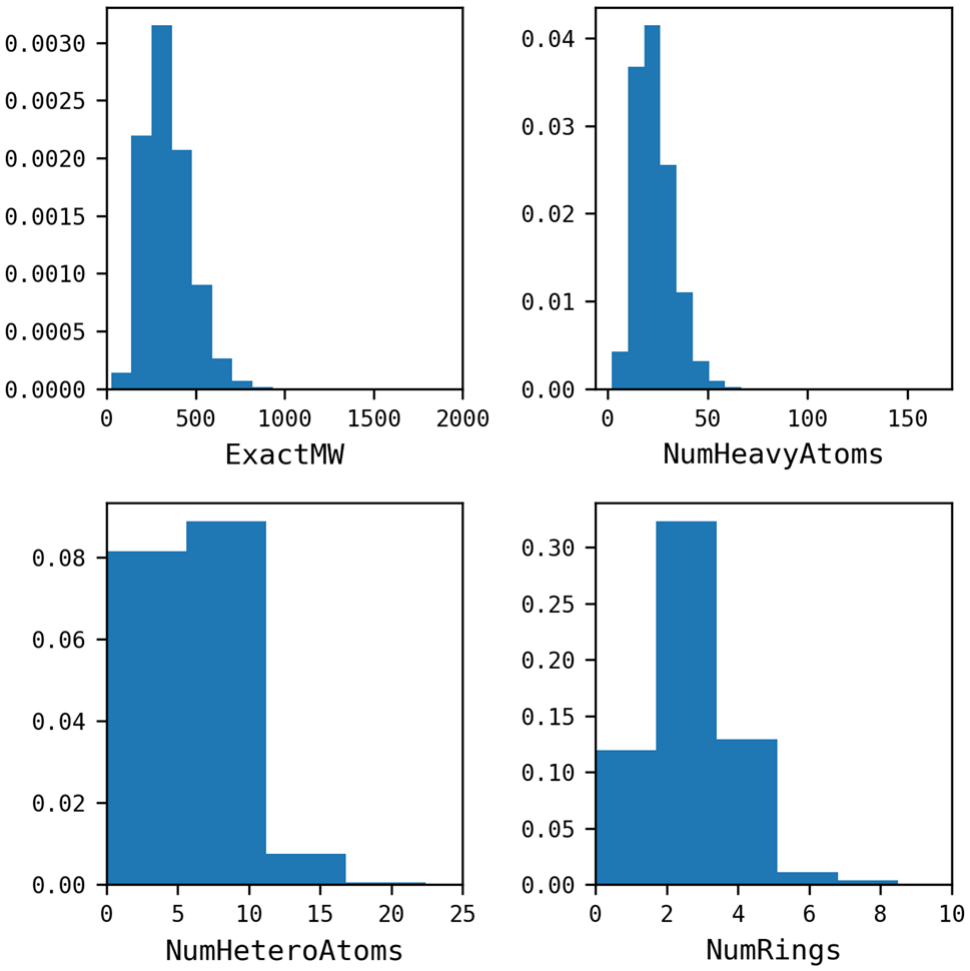
Property distributions of the starting materials extracted from the clean USPD Grants subset. Calculations were using the RDKit descriptor calculation node in KNIME

The reactions were then mapped using the Indigo Mapper node in the “Indigo Toolkit” in KNIME Analytics Platform [24] and unmapped components (for example, solvents and catalysts) were removed. Reactions were balanced as far as possible by inserting missing fragments or splitting reactions into two separate entries in the case of isomeric products, in order to achieve the same number of carbon atoms on each side of the reaction [10]. The starting material for each reaction was then extracted. Where there was more than one starting material, the one with the highest number of mapped atoms was retained, or if there was ambiguity (i.e., the same number of mapped atoms) the entry was discarded. 34,929 molecules were discarded at this stage. The properties of the starting materials were calculated by filtering out InChI Key duplicates, without considering their association with different reaction classes. 360,477 unique molecules were retained, representing a reduction of 66% of the SHREC classified reactions (Table 2). This large reduction is likely due to the nature of the data set which has been derived from the patent literature and represents lead optimisation series. The distributions of properties are plotted in normalised histograms in Fig. 5.

**Fig. 6:**
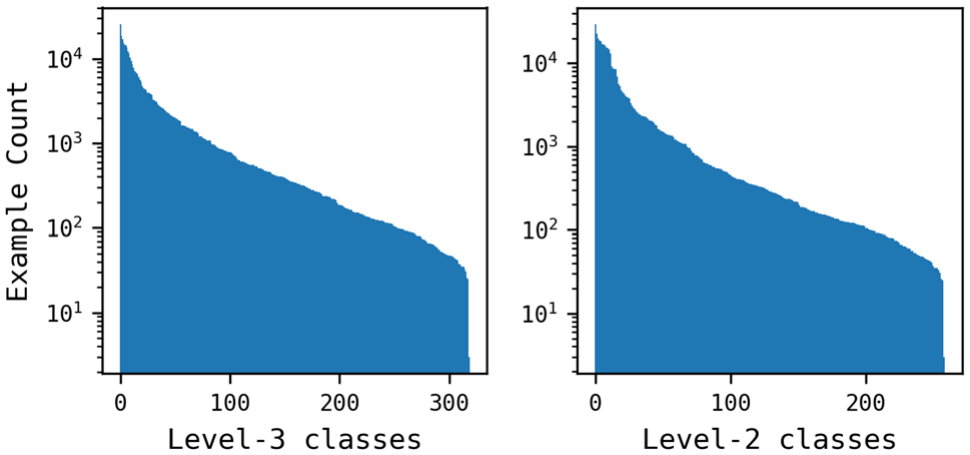
The distribution of examples for each of the reaction classes. The level-3 set consists of 319 classes and the level-2 set consists of 259 classes

The set of starting materials was then considered at different levels of the classification system with the number of classes at level-3 and level-2 yielding 319 and 259 unique reaction class labels, respectively. Duplicates from these two subsets were removed: an entry was considered a duplicate if both the starting material and the reaction class matched with another entry. The final numbers of unique entries are shown in Table 2 where it can be seen that duplicate removal (starting material and reaction class) reduced the data by 59% and 60% for level-3 and level-2 labels, respectively. A higher median number of examples per class was found for level-3 labels (342) compared to level-2 (290), although the mean values show the opposite trend (1350 and 1638 for level-3 and level-2, respectively). The distribution of examples across reaction classes at level-2 shows that some classes are more enriched compared to level-3 labels, see Fig. 6.

### Descriptors

A variety of different binary descriptors were investigated including atom, pharmacophore and functional group descriptors. These are: Avalon (1024-, 2048-, 4096-bit fingerprints); CDK Functional Group; FeatMorgan (Radius 1) (1024-bit); FeatMorgan (Radius 2) (1024-, 2048-bit); MACCS; and OChem EFG+ , as shown in Table 3. In addition to those shown in the table, we also investigated the use of the following: atom-pairs; ChemAxon Functional Groups; Chi and Kappa descriptors; Layered; Morgan; Pattern; and RDKit fingerprints, however, these all gave considerably poorer performance and so results for these are not reported here.

**Table 3:** The molecular descriptors investigated

Molecular descriptor	Description
Avalon	Hashed fingerprint mainly describing atom presences, paths, bonds, rings, and hydrogen features; implemented in the RDKit library [25]
CDK Functional Group	SMARTS-based dictionary fingerprint developed by Inte:Ligand and implemented using RDKit, which encodes the presence of 307 different functional groups [26]
FeatMorgan	Pharmacophore-based fingerprint implemented in the RDKit library, that encodes inter-distances between features and neighbour information within a defined radius [27]
MACCS	SMARTS-based implementation in the RDKit library of 166 public MACCS keys by MDL Information Systems
OChem EFG+	Integrated version of the OChem EFG fingerprint implemented using RDKit, which encodes the presence of 2080 structural features [28]

The starting materials in the level-3 and level-2 data sets were encoded as descriptors and duplicate entries were merged by appending the relevant reaction classes, as illustrated in Fig. 3. The data set sizes for the different descriptors are shown in Table 4. The different descriptors resulted in different sized data sets due to the pivoting process whereby identical descriptors labelled by different reaction classes are aggregated to a single entry. Training and test sets were generated for each data set using stratified sampling based on the number of labels associated with each entry, to give 80% training and 20% test, respectively.

**Table 4:** Data set size per descriptor-type generated from the pivoting

Molecular descriptor	Bit string length	Number of unique entries
Avalon	1024	358,648
Avalon	2048	358,765
Avalon	4096	359,019
CDK functional group	307	244,980
FeatMorgan (Binary) (Radius 1)	1024	270,049
FeatMorgan (Binary) (Radius 2)	1024	338,842
FeatMorgan (Binary) (Radius 2)	2048	338,879
MACCS	166	324,086
OChem EFG+	2080	241,230

### Multi-label classification

Multi-label classification problems are generally addressed using two alternative methods: Problem Transformation (PT), where the multi-label problem is transformed to be compatible with traditional classifiers such as Random Forests (RF) or Support Vector Machine (SVM); and Algorithm Adaptation (AA) methods (e.g. Multi-Label k-Nearest Neighbors (MLkNN)), where classifiers are modified to deal with the multi-label nature of the problem [14]. In preliminary work, not reported here, the PT method gave better performance than AA hence we focused on these methods.

PT methods can be divided into Binary Relevance, Classifier Chain, and Label Powerset. Binary Relevance (BR) simply converts each label into a binary classification problem, thus ignoring any potential correlations between labels. Classifier Chain (CC) works similarly to Binary Relevance, however, it converts the output from each binary prediction into an additional feature column that is used to produce the next predictions, thus creating a connection between the labels. Label Powerset (LP) enforces this connection further by forming a single multi-class problem by combining the original labels into all possible combinations, with each combination becoming a new derived label. LP often yields highly unbalanced data sets where some combinations of labels have very low frequencies and are associated with only a few training examples, thereby affecting prediction performance. Another issue is the potentially huge number of label-sets that can be created which can lead to memory issues. For example, a 50-class multi-label problem can potentially yield a maximum number of 2^50^ label-sets. In reality, this number is always much smaller but LP can still be very memory intensive. RAkEL (Random k-Labelsets) is an ensemble method designed to overcome potential issues related to LP [29]. RAkEL works through the construction of an ensemble of LP classifiers that are trained using smaller label-sets obtained from the random selection of *k* label subsets from the original label set. Thus, the task is computationally less demanding and the label-set distribution is less skewed.

The following problem transformation approaches were used: BR, CC and RAkEL. We used two different RAkEL methods: disjoint RAkEL (RAkELd) where the subsets of labels are non-overlapping and overlapping RAkEL (RAkELo) where overlap of the different label subsets is permitted. The use of LP was not possible due to the large number of reaction class labels. The multi-label approaches were combined with Random Forests (RF) and Support Vector Machine (SVM) classifiers using default parameters as reported in Table 5. The RAkEL methods were configured using default parameters as suggested by Tsoumakas et al. [30].

**Table 5:** Multi-label approach and classifier parameters

Classifier	Parameters
RF	n_estimators=10, criterion='gini', max_depth=None, min_samples_split=2, min_samples_leaf=1, min_weight_fraction_leaf=0.0, max_features='auto', max_leaf_nodes=None, min_impurity_decrease=0.0, min_impurity_split=None, bootstrap=True, oob_score=False, n_jobs=4, random_state=11, verbose=0, warm_start=False, class_weight=None
SVM	penalty='l2′, loss='squared_hinge', dual=True, tol=0.0001, C=1.0, multi_class='ovr', fit_intercept=True, intercept_scaling=1, class_weight=None, verbose=0, random_state=11, max_iter=1000
BR	require_dense=[True, True]
CC	require_dense=[True, True], order ()
RAkELd	labelset_size=3
RAkELo	labelset_size=3, model_count=(number of labels multiplied by 2)

### Model construction

**Fig. 7:**
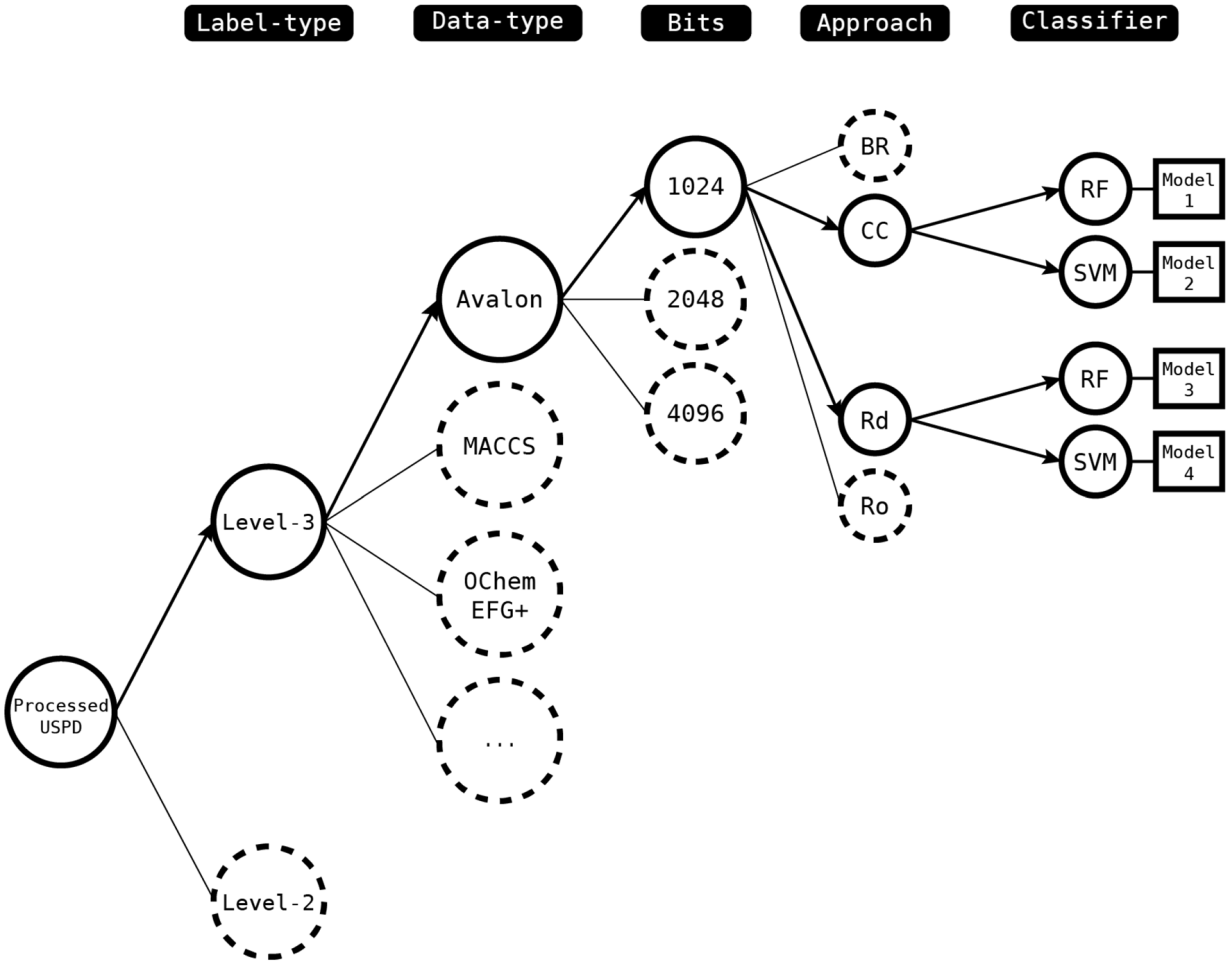
Model creation tree diagram: the bolded nodes with directed edges represent example combinations of label-type, descriptor-type, fingerprint size, multi-label approach and classifier type

Models were created by combining the different components combinatorially as shown in Fig. 7.

### Evaluation

In a binary classification problem, a given prediction is either correct or wrong, whereas in multi-label classification, it can be fully correct, fully wrong, or partially correct/wrong when some of the labels are predicted correctly but others are not. According to Tsoumakas et al. [31], two techniques are generally adopted to measure performance in multi-label classification: example-based and label-based techniques. The first is row-wise and involves comparing true and predicted labels for every example in the test set, whereas, the second is column-wise and computes metrics for each label and averages over all labels, ignoring any label dependencies. The example-based methods are most appropriate for the Reaction Class Recommender, since the quantification of individual label performance is not relevant.

The performance of the models was assessed using Recall, Precision and the F1-score, all of which can be derived from the numbers of true positives (TP), true negatives (TN), false positives (FP) and false negatives (FN), see Table 6. Recall is the ratio of true positives (TP) to the total number of positives in the test set (TP + FN), and provides a measure of the quantity of positive predictions without accounting for false positives. Precision represents the ratio of true positives to the total number of predicted positives (TP + FP), and provides a measure of the quality of the positive predictions without considering the false negatives. In multi-label classification, these two metrics are usually computed as averages using the ‘micro’ method which is based on global counts of TP, FNs and FPs (rather than calculating the values for each label independently and then averaging, which is the macro method). Micro values are preferable in the case of unbalanced data, since they avoid the issue of giving more emphasis to majority classes. The F1-score is the harmonic average of Recall and Precision and balances both factors.

**Table 6:** Performance measures using to evaluate the models

Performance measure
$${\text{Recall}} = \frac{{{\text{TP}}}}{{{\text{TP}} + {\text{FN}}}}$$
$${\text{Precision}} = \frac{{{\text{TP}}}}{{{\text{TP}} + {\text{FP}}}}$$
$$F1 = 2 \times \frac{{{\text{Recall}} \times {\text{Precision}}}}{{{\text{Recall}} + {\text{Precision}}}}$$

The selection of metrics was based on considering the intrinsic nature of the problem. In a reaction class recommendation scenario, false positives and false negatives can be weighted differently according to the needs of the final user. For example, if a user wishes to limit the output to solutions that are more reliable, the presence of a few false positives will be considered less acceptable than the presence of a few false negatives. In contrast, if the user is attempting to catch all the recommended classes while still reducing the enumeration time, the presence of a few false positives may be preferred to the presence of a few false negatives.

### Implementation details

The machine algorithms used in this project are distributed by scikit-multilearn [32] and scikit-learn [33]. These include two scikit-multilearn Problem Transformation (PT) (skmultilearn.problem_transform) approaches (BinaryRelevance and ClassifierChain) and two Ensemble Methods (skmultilearn.ensemble) (RakelO and RakelD) combined with Random Forests or Support Vector Machine classifiers (sklearn.ensemble.RandomForestClassifier) (sklearn.svm.LinearSVC) which were configured using default parameters. All descriptors were based on RDKit implementations. The multi-label approach and classifier parameters are described in Table 5.

## Results

### Model optimisation

The theoretical number of combinations of descriptor types, classification label types and machine learning approaches is 144 (nine descriptors; two sets of classification labels; four multi-label approaches; and two modelling methods). Rather than evaluate all possibilities, a staged approach was taken.

**Fig. 8:**
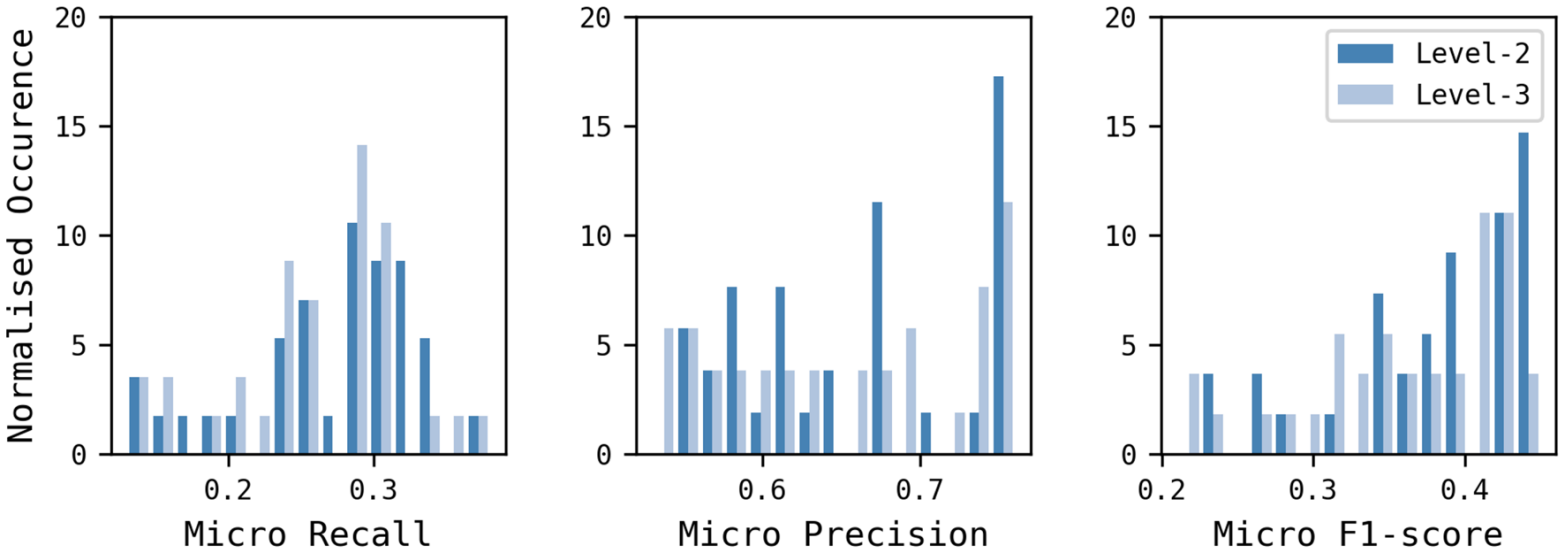
Comparison of the models built using the level-2 and level-3 classification systems. A total of 68 models are shown which vary by descriptor, multi-label approach, modelling method and classification level. The models built using the level-2 classification are in dark blue; those built using the level-3 classification are in light blue

First, the level-2 and level-3 classification levels were compared using the BR and CC multi-label classification approaches, combined with RF and SVM, and the nine different descriptors. Only 68 of the possible 72 combinations produced valid solutions. SVM failed for two descriptor/classification label configurations possibly due to a library bug. These, were OChem EFG + and the level-2 classification and FeatMorgan 1024-bit (Radius 2) and the level 3 classification, with both combinations failing for both the BR and CC approaches. Figure 8 shows the distribution of micro Recall, Precision, F1-scores as histograms for the 68 models with the level-2 classification results in dark blue and the level-3 results in light blue. The level-2 labels resulted in a higher number of best performing models according to their micro F1-scores. The best level-2 model was found for Avalon 2048-bit (micro F1-score of 0.45), whereas, the best level-3 model (micro F1-score of 0.44) was based on Avalon 4096-bit. The level-3 classification approach was chosen for the next series of experiments since it is capable of recognising a higher number of labels and because these can be generalised to level-2 labels, if required, due to the hierarchical nature of the classification scheme.

**Fig. 9:**
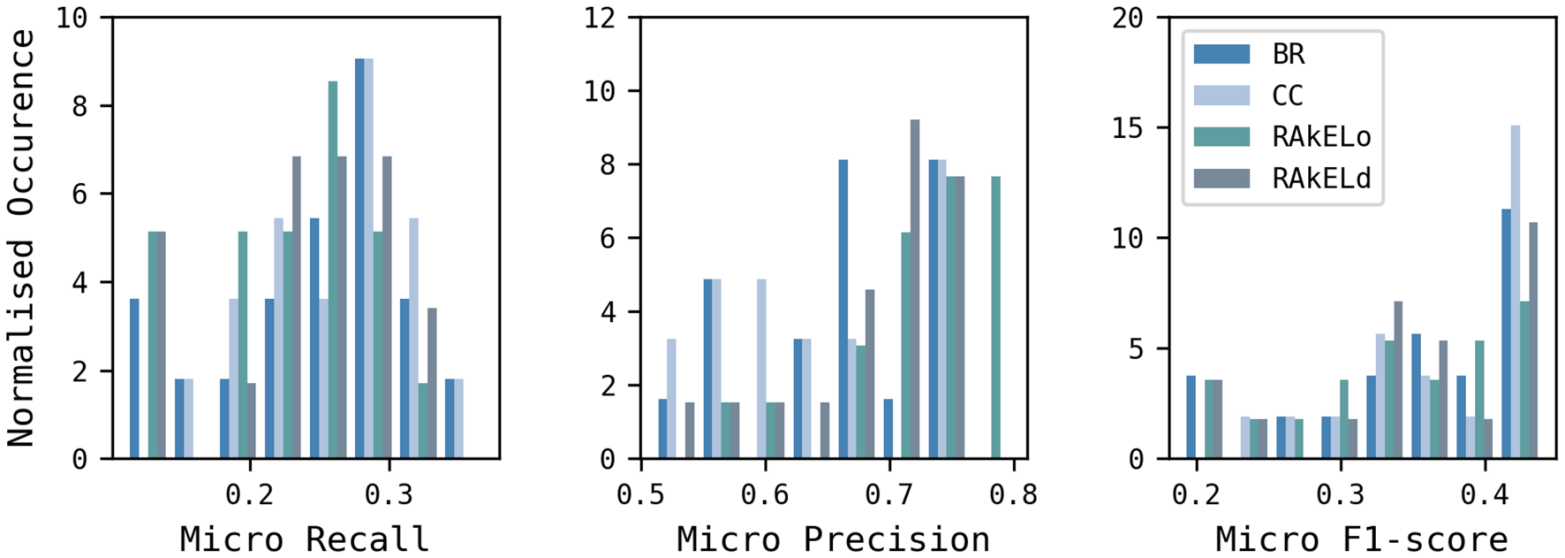
BR, CC, RAkELo, and RAkELd approaches comparison using the level-3 classification scheme

Models were then built using the level-3 data set, the nine descriptors, two PT multi-label approaches (BR, CC) and two ensemble techniques (RAkELd and RAkELo), and both RF and SVM. This time, 70 of the possible 72 models were generated; as for the previous experiment, SVM models could not be generated for FeatMorgan 1024-bit (Radius 2) for the BR and CC approaches. Micro Recall, Precision and F1-scores are shown in Fig. 9 and summarised in Table 7. The PT approaches (BR and CC) resulted in better micro Recall, while the EMs (RAkELo and RAkELd) produced better micro Precision. Table 7 also shows that CC generally resulted in better performance compared to BR, except for micro Precision and RAkELd outperformed RAkELo, possibly suggesting that the disjoint method can account for more label dependence. A one-way ANOVA analysis of the performances of the BR, CC, RAkELd, and RAkELo approaches showed that the differences were not significant at the p-value < 0.05 level for the four conditions, except for Precision where they were significant (p-value = 0.01).

**Table 7:** Performance metrics for BR, CC, RAkELo, and RAkELd approaches using RF and SVM and the level-3 data

Method	Micro recall			Micro precision			Micro F1-score		
	Min	Mean	Max	Min	Mean	Max	Min	Mean	Max
BR	0.13	0.25	0.35	0.53	0.66	0.75	0.21	0.35	0.43
CC	0.15	0.27	0.37	0.53	0.64	0.76	0.24	0.37	0.44
RAkELo	0.11	0.23	0.32	0.57	0.72	0.80	0.19	0.34	0.43
RAkELd	0.11	0.24	0.32	0.51	0.69	0.74	0.19	0.35	0.43

The memory requirements were then compared considering descriptor type and multi-label approach, (Table 8). Classifier type was not discriminated at this stage; hence the reported values represent the maximum amounts required by the most memory-consuming classifier per configuration during the model training. CC was selected as the multi-label classification method for the subsequent experiments since the performance was marginally better than BR (Table 7) and the memory requirements were lower than for the RAkEL methods (Table 8).

**Table 8:** Comparison of maximum memory requirements for the PT multi-label approaches BR and CC and the ensemble approaches for the different descriptors

Molecular descriptors	Features	Maximum memory request (GB)	
		BR/CC	RAkELo/RAkELd
Avalon	1024	15.3	16.6
Avalon	2048	26.4	27.6
Avalon	4096	48.7	49.8
CDK Functional Group	307	5.8	10.1
FeatMorgan (Binary) (Radius 1)	1024	11.6	16.3
FeatMorgan (Binary) (Radius 2)	1024	13.4	16.0
FeatMorgan (Binary) (Radius 2)	2048	24.8	25.3
MACCS	166	5.6	9.1
OChem EFG+	2080	18.3	21.2
Average		18.8	21.3

**Fig. 10:**
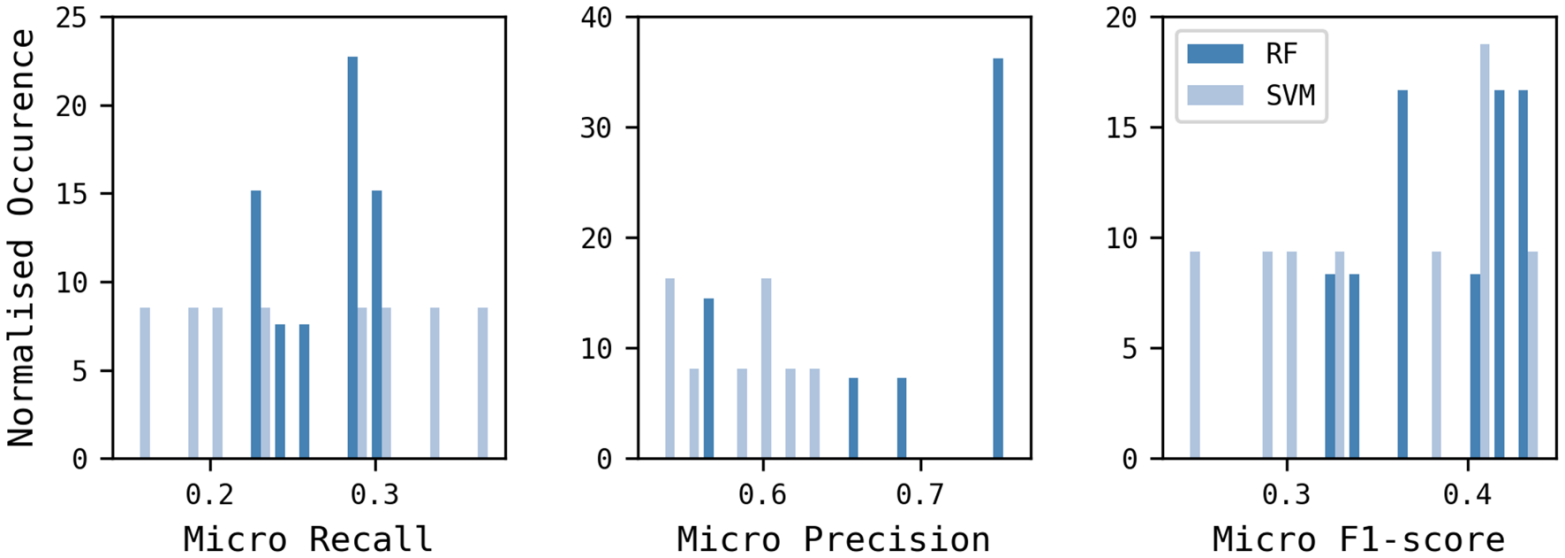
Comparison of RF and SVM, across the nine descriptors, using CC and the level-3 classification scheme

Next, the machine learning methods RF and SVM were compared over the nine descriptors, using CC and the level-3 classification scheme, as shown in Fig. 10 and Table 9, where the results are sorted on micro F1. The FeatMorgan 1024-bit (Radius 2) CC-SVM model does not appear among the results since the SVM classifier did not work with this descriptor using level-3 labels. The RF models generally performed better than the corresponding SVM models based on the F1 score, with the average values across nine and eight models, respectively, corresponding to 0.39 and 0.35, respectively. RF was therefore chosen as the machine learning approach. Another factor favouring the use of RF is that the scikit-learn implementation of RF supports multi-threading making it significantly quicker than SVM, which is an important factor to bear in mind given the intended use of the model in iterative de novo design.

**Table 9:** Level-3 CC-RF model performance metrics

Fingerprint	Setup	Micro recall	Micro precision	Micro F1-score
Avalon 4096-bit	CC-RF	0.31	0.75	0.44
Avalon 4096-bit	CC-SVM	0.37	0.53	0.44
Avalon 2048-bit	CC-RF	0.30	0.75	0.43
Avalon 1024-bit	CC-RF	0.29	0.76	0.42
FeatMorgan 2048-bit Radius 2	CC-RF	0.29	0.75	0.42
Avalon 2048-bit	CC-SVM	0.33	0.54	0.41
FeatMorgan 1024-bit Radius 2	CC-RF	0.29	0.75	0.41
FeatMorgan 2048-bit Radius 2	CC-SVM	0.31	0.6	0.41
Avalon 1024-bit	CC-SVM	0.29	0.56	0.38
MACCS	CC-RF	0.25	0.69	0.37
OChem EFG+	CC-RF	0.26	0.57	0.36
FeatMorgan 1024-bit Radius 1	CC-RF	0.23	0.66	0.34
CDK Functional Group	CC-RF	0.23	0.57	0.33
OChem EFG+	CC-SVM	0.23	0.63	0.33
FeatMorgan 1024-bit Radius 1	CC-SVM	0.20	0.60	0.30
MACCS	CC-SVM	0.19	0.59	0.28
CDK Functional Group	CC-SVM	0.15	0.62	0.24

Comparing the different descriptor types, the hashed fingerprints were more effective than dictionary-based fingerprints with Avalon and FeatMorgan giving the best performance followed by MACCS, OChem EFG + , and CDK Functional Groups. The best performance according to the F1-score was found for Avalon 4096, with its 2048- and 1024-bit versions performing similarly and being preferred due to their significantly lower memory requirements (Table 8). FeatMorgan Radius 2 produced better models than FeatMorgan Radius 1, possibly due to the wider environment encoded by the descriptors.

MACCS also produced one useful model (i.e., CC-RF) which is surprising considering that the multi-label classification guidelines suggest to use a number of features higher than the number of labels to be predicted by the model [34]. In addition, MACCS models required a maximum of 5.6 GB of memory in the final model validation (Table 8) which is significantly lower than any of the other descriptors. OChem EFG + combined with RF had similar performance to MACCS, but required 3.3 times the amount of memory. CDK Functional Groups resulted in the worse performing models. This is possibly due to the low number of features and therefore the lower discriminative ability compared to OChem EFG + . On the basis of performance and memory requirements, Avalon 1024-bit CC-RF and MACCS CC-RF were selected for further evaluation.

## Validation

The Reaction Class Recommender is aimed at reducing the number of product molecules that are generated during de novo design while improving the synthetic accessibility of the designed molecules. The first validation experiment investigates the extent to which the Reaction Class Recommender is able to reproduce the known reaction classes for a set of reactions extracted from the literature. The second validation compares the performance of single step de novo design with, and without, the use of the Reaction Class Recommender. The final experiment demonstrates the use of the recommender in a retrospective de novo design setting based on known drugs.

### Journal of Medicinal Chemistry (JMC) class prediction

The Reaction Class Recommender was applied to a set of starting materials extracted from the reaction literature for which the reaction classes had been determined using SHREC. The recommended reaction classes were compared with the annotated classes and the level of agreement determined. One of three possible outcomes was recorded for each starting material: if the recommended classes contained the annotated class, then the recommender was considered to be correct; if the recommended classes did not contain the annotated class, the recommender was considered as wrong or incorrect; if the recommender did not make any recommendations, then no-recommendation was recorded. (Note that multi-label approaches are essentially ensembles of binary classifiers and do not return any label if none of the binary classification tasks return a positive result. This differs from multi-class methods which will assign a label to one or more classes.) The number of recommendations made for each starting material was also recorded.

A set of 26,757 single-step reactions was extracted from Journal of Medicinal Chemistry (JMC) publications covering the period January–September 2018, using Reaxys. The reactions were cleaned using the same protocol as applied to the USPD (24,606 were retained); reaction vectors were calculated; the reactions were classified using SHREC and all entries associated with a high prediction probability were retained, based on a minimum credibility score of 0.25 as described in Ghiandoni et al. [13]. 16,582 entries remained at this stage. The reaction classes were represented at level-3 of the SHREC hierarchy and duplicates, that is, identical starting materials and identical class labels, were filtered out leaving 11,544 entries. The chemical structures were then checked to verify their integrity by converting them to RDKit objects and reconverting them to SMILES. A total of 11,539 reactions were retained.

**Fig. 11:**
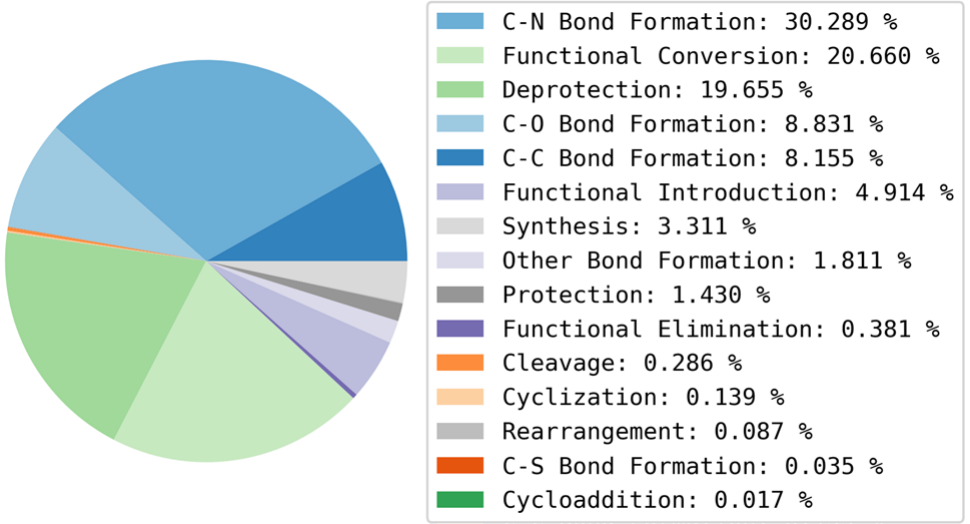
Level-1 reaction classification of the JMC data set

**Fig. 12:**
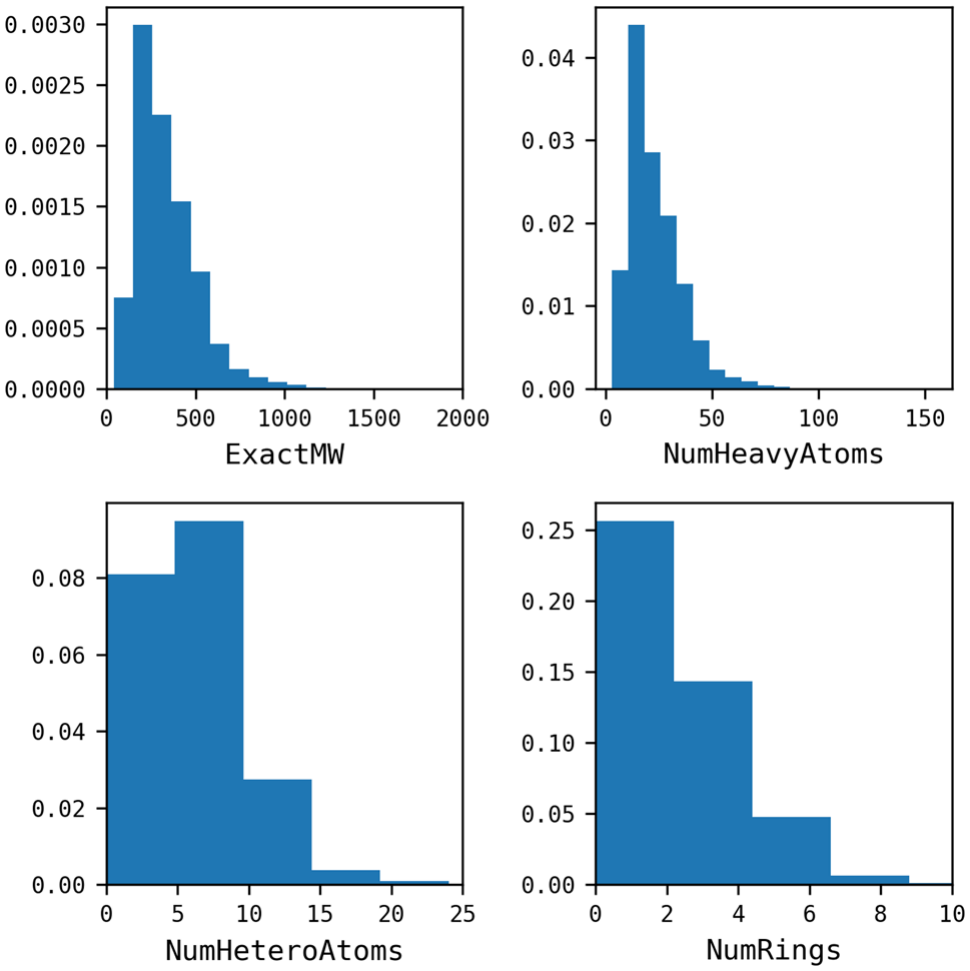
Property distribution of the starting materials extracted from the classified JMC 2018 test set

The data set was also described by level-1 reaction class labels and its reaction class composition was compared with the USPD Grants data set used to train the Reaction Class Recommender. The class composition is reported in Fig. 11 which shows that the composition of reaction classes is similar to that of the training data. The distributions of the physicochemical properties of the starting materials are shown in Fig. 12 and are also broadly comparable with the training data.

Reaction Class Recommenders trained on the USPD using two different descriptors (Avalon 1024-bit and MACCS fingerprints) were applied to the JMC data and evaluated at different levels of the classification hierarchy (i.e., level-3, 2 and 1). Results (correct, wrong and no-recommendation), expressed as percentages, are reported in Table 10. The average numbers of recommendations per starting material are 1.9 (3.4 excluding the starting materials returning no-recommendation) and 2.7 (4.4 excluding the starting materials returning no-recommendation) for the Avalon 1024-bit and MACCS recommenders, respectively. Although the percentage of examples for which no recommendation is made may appear to be high, these represent examples that fall outside of the domain of the recommender, and in a de novo design setting, this would simply mean that no filtering on the reaction vectors should be applied.

**Table 10:** Performance of the Avalon and MACCS Reaction Class Recommenders on the JMC data expressed as percentages

Model	Label-type	Correct	Wrong	No-recommendation
Avalon 1024-bit	Level-3	33.5	21.9	44.6
	Level-2	34.4	21.0	44.6
	Level-1	41.9	13.5	44.6
MACCS	Level-3	37.1	23.1	39.8
	Level-2	38.0	22.2	39.8
	Level-1	45.0	15.2	39.8

The MACCS Reaction Class Recommender resulted in higher percentages of both correct and wrong recommendations compared to the Avalon Reaction Class Recommender which, in contrast, returned a higher percentage of starting materials for which no recommendation was made. This finding is consistent with the lower compression rate seen for Avalon descriptors in the training data whereby the starting materials are generally associated with fewer reaction classes compared to those seen for the MACCS descriptors. Moving up the levels in the classification hierarchy does not change the percentage of starting materials for which no recommendations were made, however, it does increase the chance of matching the correct label due to the reduction in the total number of classes that could be matched. Although this may suggest that the Reaction Class Recommender is more accurate, it is not as useful from the perspective of de novo design since the broader reaction classes encompass a large number of more specific reaction classes so that fewer reaction vectors would be filtered out.

**Fig. 13:**
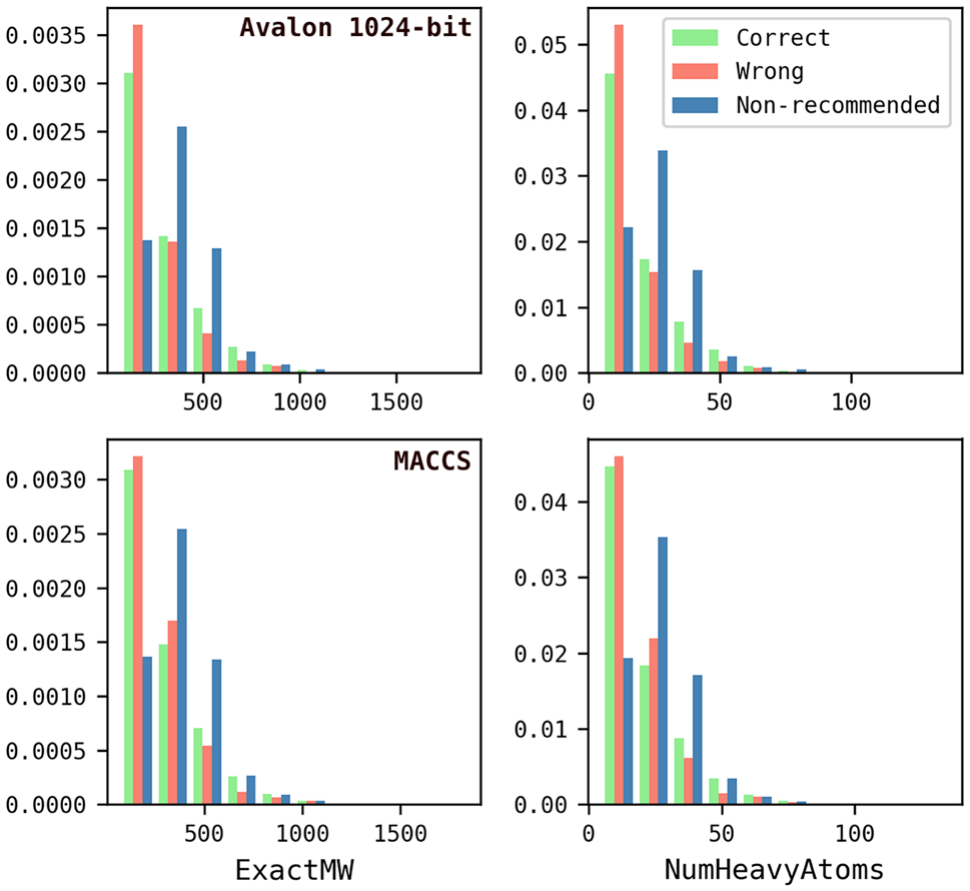
Property distributions for the starting materials coloured by correct (green), wrong (red), and no-recommendation (blue) following application of the Reaction Class Recommenders

The Reaction Class Recommenders were further analysed by plotting the percentages of correct, wrong, and no-recommendation entries for different property ranges of the starting materials, Fig. 13. The relative percentages of correct and wrong entries are broadly similar across the property ranges and both decrease as the molecules become larger. However, the percentage of no-recommendation entries increases as the molecule size increases. This can be rationalised by comparing these distributions with the training data properties reported in Fig. 6. As the test starting materials start to move away from the domain of the training set, the algorithm tends to be unable to make recommendations for them. In particular, both models maintain low percentages of no-recommendations in fragment-like space (MW < 300), however, the ratio of no-recommendations increases at higher molecular weights presumably due to the sparsity of starting materials with higher molecular weights in the training data.

The Reaction Class Recommenders were also analysed at the most general class labels (i.e., level-1) to determine whether wrong predictions and no-recommendations were more frequent for some reaction classes. The absolute numbers of wrong predictions and no-recommendation entries per class were divided by the total numbers of examples in the data set to obtain normalised statistics as shown in Table 11 along with the total numbers of examples per class. Comparing these results with the proportions of level-1 reaction classes in the training data reported in Fig. 4 shows that the reaction classes that generally result in higher numbers of wrong predictions are those that are less frequent in the training data, or they represent reaction classes with greater ambiguity. For example, “Deprotection” and “Functional Conversions” are similarly frequent in the training set (~ 13% and 20%, respectively) but there are almost 7 times as many errors for “Functional Conversions” compared to “Deprotection” for the MACCS Reaction Class Recommender (0.03 and 0.2, respectively). This can be rationalised by protecting groups being easily identified by molecular descriptors, whereas functional groups can be present in molecules for different purposes other than the intended reaction, for example, they may be responsible for biological activity in the final structures. The ratios of no-recommendation entries are broadly similar across the two Reaction Class Recommenders.

**Table 11:** Ratios of wrongly predicted entries and entries for which no recommendation was made for each reaction class at level-1 of the hierarchy for both Reaction Class Recommenders

Class	Total examples	No-recommendation ratios		Wrong prediction ratios	
		Avalon 1024-bit	MACCS	Avalon 1024-bit	MACCS
C–C bond formation	941	0.40	0.32	0.16	0.11
C–N bond formation	3495	0.50	0.47	0.13	0.11
C–O bond formation	1019	0.32	0.32	0.19	0.18
C–S bond formation	4	0.25	0.50	0.50	0.75
Cleavage	33	0.82	0.85	0.06	0.12
Cyclization	16	0.19	0.25	0.31	0.44
Cycloaddition	2	0.50	0.50	0.50	0.00
Deprotection	2268	0.52	0.42	0.05	0.03
Functional conversion	2384	0.40	0.35	0.22	0.2
Functional elimination	44	0.41	0.50	0.36	0.39
Functional introduction	567	0.35	0.31	0.18	0.20
Other bond formation	209	0.52	0.50	0.22	0.21
Protection	165	0.27	0.26	0.37	0.42
Rearrangement	10	0.20	0.50	0.50	0.80
Synthesis	382	0.38	0.34	0.23	0.21

The Reaction Class Recommenders were further compared by examining the intersection of the wrong predictions. The Avalon 1024-bit Reaction Class Recommender and the MACCS Reaction Class Recommender resulted in 2531 and 2661 wrong predictions, respectively, of which 1585 are shared (63% and 60% of the total number of wrongly predicted entries). This relatively high intersection indicates a strong relationship between the two Reaction Class Recommenders, however, the percentages of non-shared wrong predictions suggest that the two Reaction Class Recommenders treat some of the test data in different ways.

**Fig. 14:**
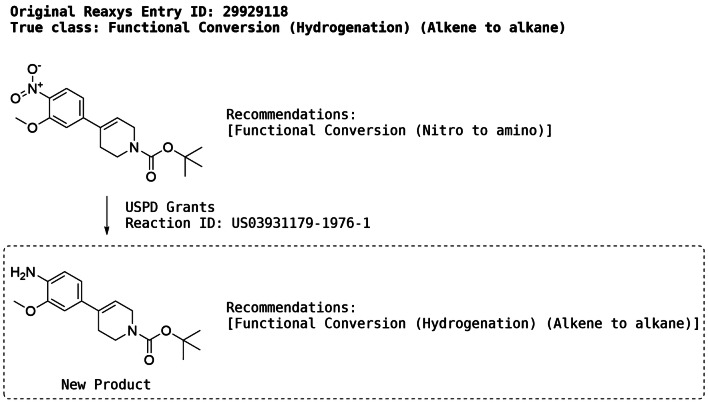
Analysis of a *wrong* recommendation using the CC-RF MACCS model. The recommender did not suggest the *correct* class associated with the top molecule. However, application of the suggested transformation produces a new product for which the correct class is predicted

The wrong predictions were further analysed by manual inspection and revealed that although the true reaction classes were missing in the recommended classes, most of the entries actually received meaningful recommendations from both models. An example of a meaningful but “wrong” recommendation is shown in Fig. 14. The annotated class for the top molecule is Functional Conversion (Hydrogenation) (Alkene to alkane) whereas the recommended class is Functional Conversion (Nitro to amino) which is, therefore, labelled as wrong. However, application of reaction vectors consistent with the recommended class (Nitro to amino) produces the bottom molecule for which the recommender outputs the hydrogenation reaction. Thus, although the first application of the recommender is “wrong” with respect to the annotated class, the sequential application of the recommender can still drive the algorithm towards the selection of appropriate reaction classes even if the correct recommendations are not produced in the first round. The underlying reason for this behaviour is the increased frequency of hydrogenations in the presence of amine groups relative to hydrogenations in the presence of nitro groups within the training data.

### Single-step de novo design

The use of the Reaction Class Recommender for single step de novo design was evaluated using our reaction-based de novo design method which is described in the Introduction. A de novo design workflow was constructed consisting of a set of fragments as starting materials, a set of reagents and a set of reaction vectors. A control experiment was run first which consisted of a full enumeration without the use of the Reaction Class Recommender. The experiment was then rerun using the Reaction Class Recommender with the recommended reaction classes acting as a filter on the reaction vectors, so that only those belonging to the recommended classes were considered for de novo design. The two experiments were compared on the number of products obtained, the synthesisability of the products and execution time.

Twenty six fragments were selected from the commercial screening library DSPL [35, 36] as starting materials. Each of these was known to reproduce one or more compounds for which activity data is reported in the ExCAPE database [37], i.e., every starting material was known to be the precursor of an active compound therein. The starting materials were restricted to those for which recommender produced recommendations and for which the known active could be generated using reaction classes that the recommender was trained on. A set of reagents was selected from the Sigma-Aldrich commercial catalogue as a source of reagents. The reaction vectors consisted of a set of 11,545 unique reaction vectors which were classified using our classification model with 46% receiving classification labels.

The starting materials were described by MACCS fingerprints and the MACCS Reaction Class Recommender was used to make recommendations for each starting material. The recommendations were made at level-3, and then analysed at levels 3, 2 and 1 by moving through the classification hierarchy. Table 12 shows that moving up the hierarchy to more general reaction classes reduces the mean number of recommendations per starting material. However, this generalisation actually increases the number of applicable reactions rather than reducing it, as discussed above. For example, the level-3 recommendations of “C–C Bond Formation (Coupling) (Suzuki)” and “C–C Bond Formation (Coupling) (Heck)”, would result in the application of reaction vectors that fall within their reaction sub-classes only (six reaction classes in total); whereas the overarching level-1 reaction class “C–C Bond Formation” includes 56 types of “C–C Bond Formation” reaction classes. Hence, the use of more general labels is expected to increase the number of applicable reaction vectors, and therefore the size of the product library that is generated.

**Table 12:** The minimum, maximum, mean and median number of recommended classes per starting material for the different classification levels along with the numbers of applicable reaction vectors

Classification level	Number of recommended classes				Number of applicable reaction vectors	
	Minimum	Mean	Median	Maximum	Absolute number	Relative value
Level 3	1	2.15	1.5	12	85	1.0
Level 2	1	2.08	1.5	11	94	1.1
Level 1	1	1.46	1	3	170	2.0
Control	–	–	–	–	357	4.2

The Control represents a full enumeration (without the use of the Reaction Class Recommender)

The de novo design tool was then run in the four different modes (Control and using the Reaction Class Recommender at the three different classification levels) for the 26 starting materials. Each of the four product libraries was analysed as follows. The total number of unique products was determined by filtering out InChI Key duplicates; the percentage overlap of the generated compounds with ExCAPE was determined by comparing the library InChI Keys with the ExCAPE InChI Keys; and average synthetic accessibility estimates were calculated using RSynth [38] and SAscore [39]. In addition, the time required to enumerate each product library was recorded.

Results are summarised in Table 13 where it can be seen that the recommended and control pipelines differ substantially in the number of unique products generated. The level-3 and level-2 recommendations produced similar numbers of products, whereas the level-1 and control pipelines generated collections that are 1.68 and 3.23 times larger than the level-3 library, respectively. Table 13 also shows decreasing enumeration times as the specificity of the labels increases, and demonstrates that the use of the recommender speeds up the design process, with the level-3 recommender taking approximately one third of the time of the control experiment. All of the Reaction Class Recommender pipelines present higher percentage overlap with ExCAPE compared to the control pipeline. This enrichment of known compounds is evidence of the ability of the Reaction Class Recommender to suggest reaction classes that are more likely to be applied in reality.

**Table 13:** Library statistics for recommended and control pipelines

	No. of unique products	% Overlap with ExCAPE	Mean RSynth	Mean SAscore	Time (s)
Level 3	43,952	0.22	0.57	1.69	381.22
Level 2	49,988	0.20	0.56	1.78	412.47
Level 1	73,741	0.19	0.54	1.83	585.03
Control	141,834	0.12	0.52	1.80	991.65

Average RSynth and SAscore across the libraries are also shown in Table 13. The RSynth scores range between 0 and 1, where higher values mean higher accessibility; whereas, the SAscores range between 1 and 10, with higher values representing lower accessibility. For both scores, as the specificity of the labels increases, the average synthetic accessibility increases. Note that, although the libraries generated with the use of the recommender correspond to subpopulations of the control library, they all describe a clear shift toward higher synthetic accessibility values. An independent-samples t-test was conducted to compare RSynth and SAscore in the control (M_RSynth_ = 0.524, SD_RSynth_ = 0.201, M_SAscore_ = 2.291, SD_SAscore_ = 0.333) and recommended libraries (Level-1 (M_RSynth_ = 0.543, SD_RSynth_ = 0.190, M_SAscore_ = 2.227, SD_SAscore_ = 0.301), Level-2 (M_RSynth_ = 0.563, SD_RSynth_ = 0.033, M_SAscore_ = 2.177, SD_SAscore_ = 0.280), Level-3 (M_RSynth_ = 0.571, SD_RSynth_ = 0.181, M_SAscore_ = 2.173, SD_SAscore_ = 0.283)). A significant difference (p-value < 0.0001) in the scores was found for all pair-wise comparisons at a confidence level of 95%. The effect sizes for these analyses were found to exceed Cohen’s [40] convention for a small effect (d = 0.20) except for the control-level-1 which reported Cohen’s d_RSynth_ lower than 0.20.

**Fig. 15:**
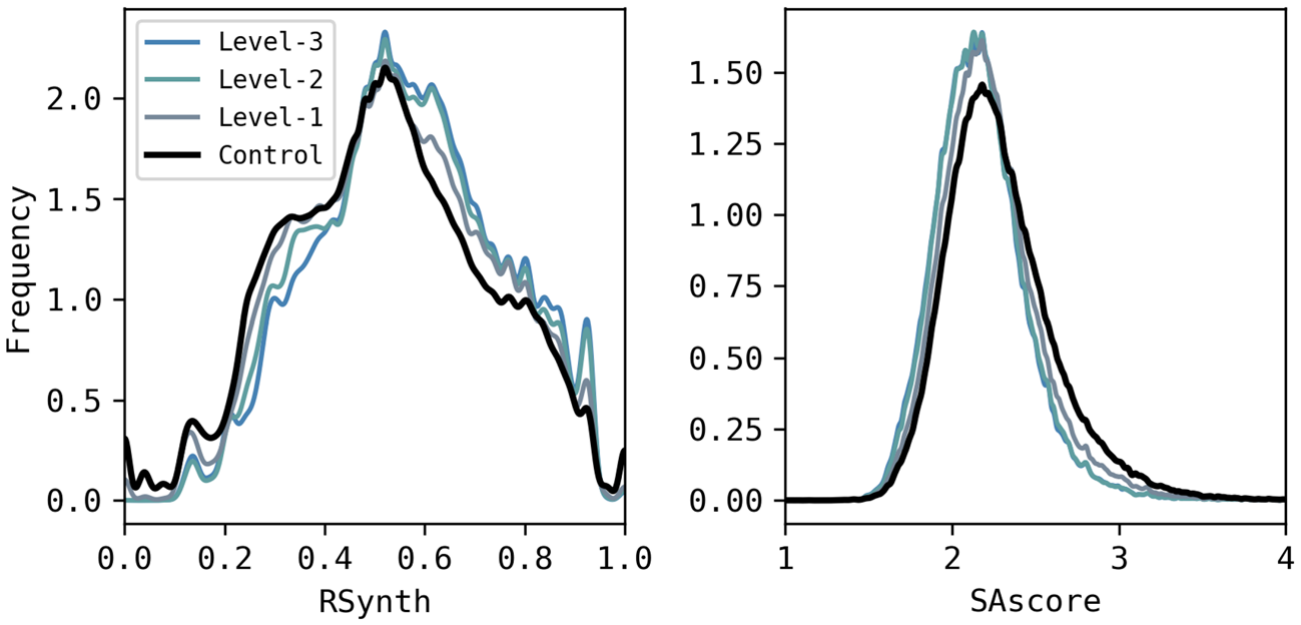
RSynth and SAscore distributions per library

The RSynth and SAscore values are plotted as overlapping density plots in Fig. 15 which show the shifts in synthetic accessibility scores towards improved values.

**Fig. 16:**
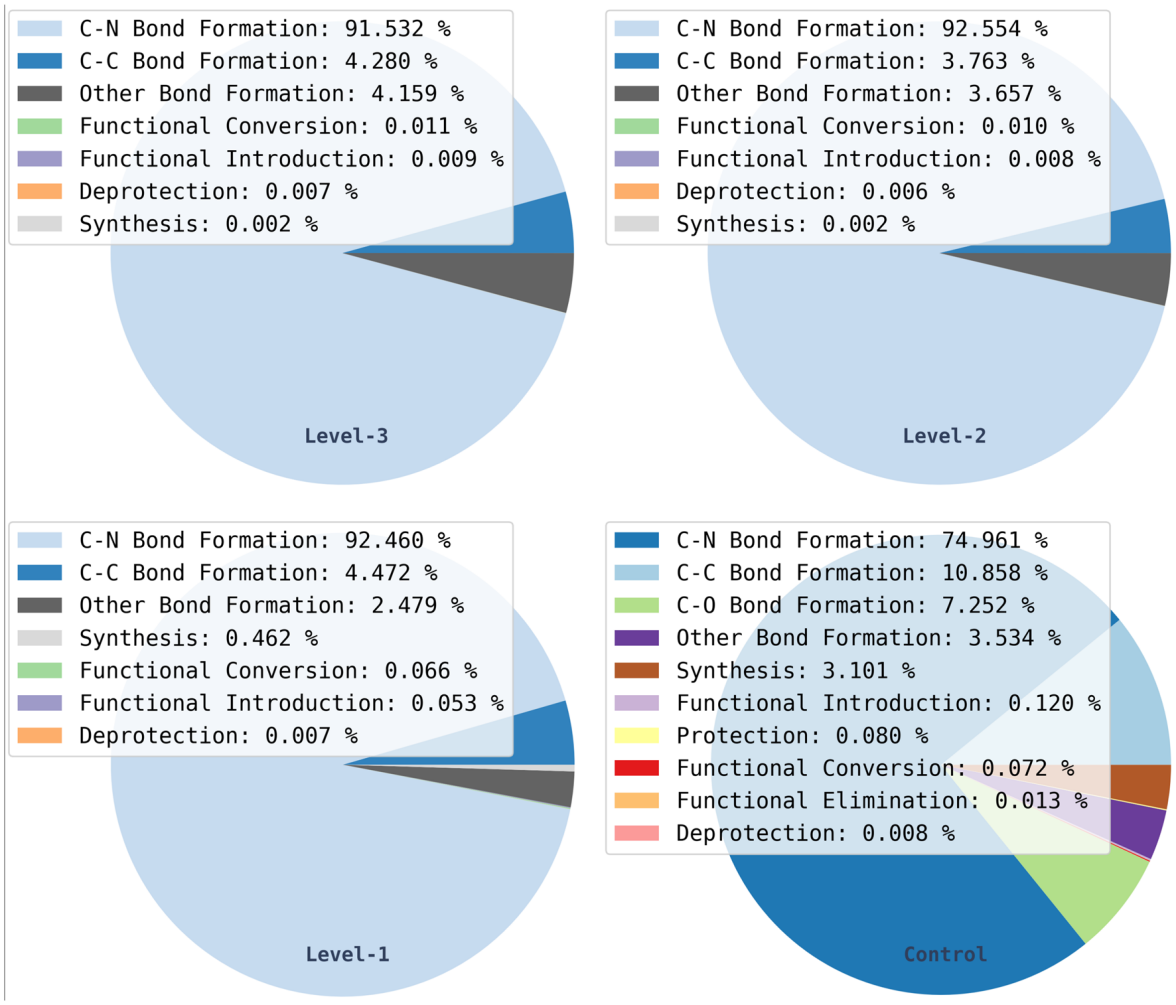
Level-1 reaction class distributions across libraries

The product libraries were then analysed by reaction class composition in order to determine possible effects of the Reaction Class Recommender on the distributions of reaction classes. Results for the level-1 reaction classes are shown as pie charts in Fig. 16.

The distributions of reaction classes at level-1 of the hierarchy are similar for the three Reaction Class Recommender experiments, whereas there is a wider variety of classes in the control experiment. More specifically, “C–O Bond Formation”, “Functional Elimination”, and “Protection” are not present at all in the recommended libraries indicating that these classes were not suggested for any of the starting materials. Although 7 of the 26 starting materials presented functional groups suitable for “C–O Bond Formation” reactions, this reaction class was not recommended for any of these structures. This can be explained by a lack of related examples in the training data, see Fig. 4, although, interestingly, the rarer class “Other Bond Formation” was recommended for three of the starting materials. “Functional Elimination” and “Protection” were not recommended at all, possibly because of the low functionalisation of the starting materials or because of the lack of examples in the training data.

**Fig. 17:**
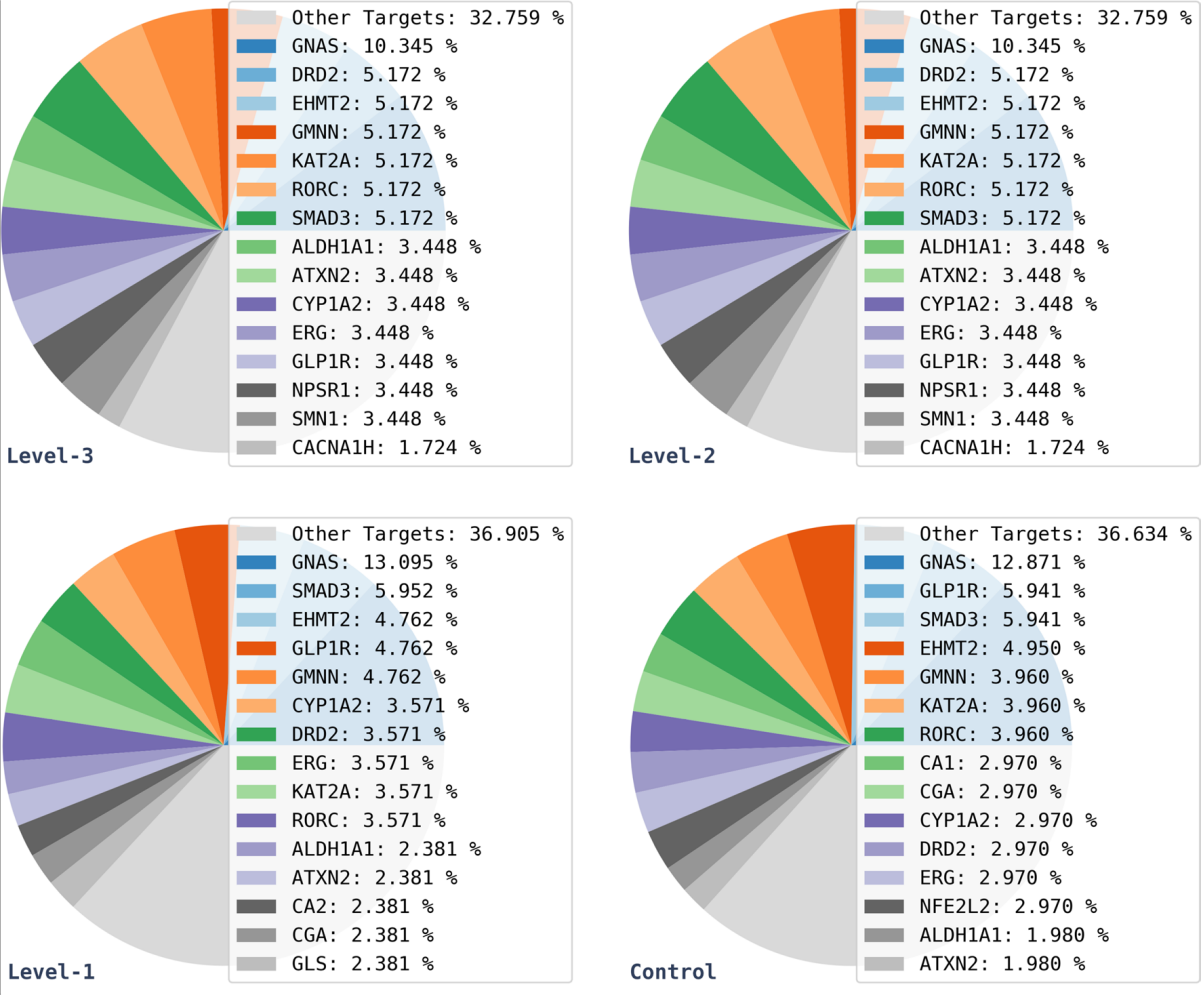
Target hits distributions across libraries

The libraries were analysed further by evaluating the top 25 biological targets associated with the reproduced active molecules in each data set, to examine possible effects of the Reaction Class Recommender on the distribution of target hits. The target information was extracted from ExCAPE. Results are reported in Fig. 17 which shows that the level-3 and level-2 distributions are identical, thus indicating the presence of the same actives in the two data sets, whereas the level-1 and control distributions report slightly different distributions including a higher percentage of “Other Targets”, indicating that the application of more reaction classes possibly expanded the target coverage. This hypothesis was verified by determining the number of unique targets hit per library which increased from 16 targets for the level-3 and level-2 libraries to 40 and 44 targets for level-1 and control libraries, respectively.

### Retrospective de novo design

The use of the Reaction Class Recommender was further assessed in retrospective reaction-based de novo design aimed at rediscovering a set of small-molecule drugs. The de novo design scenarios were compared with and without the use of the Reaction Class Recommender with the analysis focusing on the size of the chemical space explored along with the ability to rediscovery the known drugs.

The validation consists of an in-house pseudo-retrosynthetic framework called RENATE (i.e., Retro synthEtic desigN using reAcTion vEctors) which is similar to that implemented in Flux by Fechner and Schneider [41, 42]. Flux cleaves a reference ligand into fragments by applying the RECAP bond cleavage rules. It then searches a database of fragments which was also created using the RECAP rules and replaces fragments in the reference with examples in the database with similar attachment points.

In RENATE, a reference molecule is fragmented in a similar way to Flux (here using the BRICS implementation in RDKit) to form a set of reference fragments, using a minimum fragment size of five heavy atoms. The reference fragments are sorted on number of attachment points and then number of atoms. The fragment at the top of the ranked list is identified as the scaffold or starting material, with the remaining fragments considered as substituents or reagents. This heuristic ensures that the structure generation starts from highly connected fragments in order to build candidates from ‘the inside outside’. For each reference fragment, similar fragments are retrieved from a database of commercial reagents. The new fragments are then combined as follows. In the first iteration, RENATE combines the fragments retrieved for the starting material with those retrieved for the next fragment in the ranked list. For example, the sets {*a*_1_, *a*_2_, …, *a*_x_} and {*b*_1_, *b*_2_, …, *b*_y_} are combined combinatorially: for each pair of fragments (one from set *a* and one from set *b*), the set of reaction vectors is searched and a product is generated for each applicable reaction vector. Hence, a set of product molecules is produced (e.g. {a_1_–b_3_, b_2_–a_5_, a_9_–b_11_}) which are scored and the top scoring products form a new set of starting materials. These are then input to the structure generator to be combined with the next fragment set (e.g. {c_1_, c_2_, …, c_z_}). The main difference compared to Flux, is the use of real reagents and real reactions (as defined by reaction vectors) in the assembly step. Note that the rediscovery of the drug is not guaranteed using this setup since this requires the appropriate fragmentation of ligands using BRICS, and the presence of appropriate reagents and reaction vectors.

**Fig. 18:**
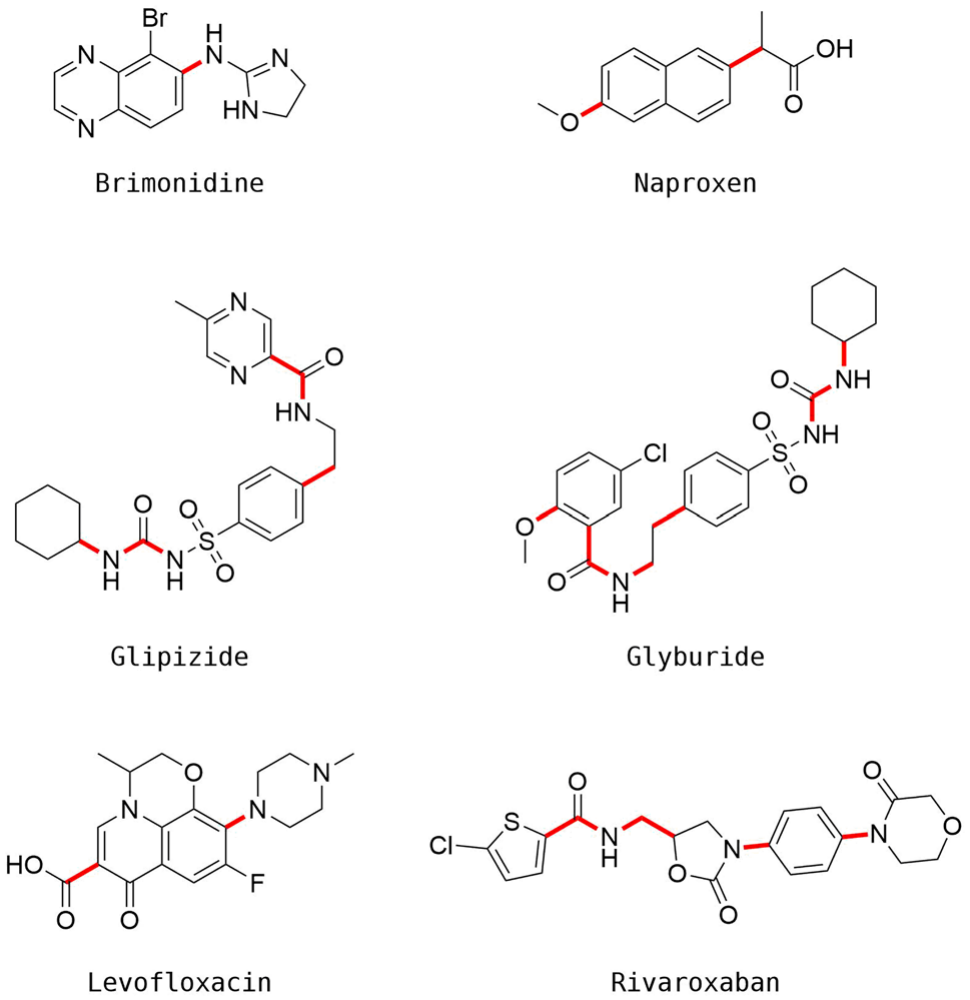
The small-molecule drugs selected for the retrospective validation

A set of six drugs was selected which were known to be correctly fragmented and regenerated by RENATE, as shown in Fig. 18. The reagent set consisted of a set of 746,245 cleaned building blocks (sanitised using RDKit, neutralised and duplicates removed) provided by Enamine [43] and the reaction vectors consisted of a set of 92,530 derived from the USPD Grants reactions (these are a subset of the 115 K unique classified reaction vectors described in Ghaindoni et al. [13] which could processed correctly by the structure generation algorithm). Each drug was fragmented, the top 1000 reagents were selected for each reference fragment (based on RDKit Morgan count fingerprints (1024-bit radius 2) and Euclidean distance) and the reagents were assembled into product molecules using the reaction vectors as described above. The final products were scored on similarity against the reference drug, also using RDKit Morgan count fingerprints (1024-bit radius 2) and Euclidean distance.

RENATE was first run for each drug without the Reaction Class Recommender to determine the total number of products generated. It was then run with the Avalon 1024-bit Reaction Class Recommender configured to produce level-3 reaction class recommendations which were used to filter the reaction vectors that were applied. Note that when the recommender was unable to make recommendations, then no filter was applied. The total number of products generated with the recommender was recorded along with the enumeration times.

Results are shown in Table 14. In all cases, the known drug was regenerated successfully for the runs without, and with, the use of the Reaction Class Recommender. In all cases, the recommender led to a significant reduction in the number of products generated (i.e., solutions explored by the algorithm) and enumeration times, suggesting that it provides a much more efficient management of the computational resources while preserving the chance of finding relevant molecules. The mean reduction in generated products and enumeration times correspond to 62% and 50%, respectively.

**Table 14:** Results from the retrospective validation

Drug	Steps	Number of products generated		Enumeration times (h)	
		Without recommender	With recommender	Without recommender	With recommender
Brimonidine	1	333,361	97,842 (− 71%)	3.0	1.2 (− 60%)
Glipizide	2	732,705	251,821 (− 66%)	5.2	2.5 (− 52%)
Glyburide	2	1,317,776	1,016,319 (− 23%)	7.5	6.3 (− 16%)
Levofloxacin	1	732,285	135,084 (− 82%)	4.1	1.5 (− 63%)
Naproxen	1	425,693	113,726 (− 73%)	3.1	1.3 (− 58%)
Rivaroxaban	3	1,282,308	536,212 (− 58%)	7.7	3.9 (− 49%)

Each ligand is described in the number of steps required for its regeneration, number of products generated by the algorithm and enumeration times, without and with the use of the Reaction Class Recommender

## Conclusions

Reaction-based de novo design aims to address synthetic accessibility by applying structural transformations that occur in real synthetic routes. Reaction vectors provide an automated way of extracting such transformation rules from reaction databases and when coupled with a structure generation method they provide a highly customisable de novo design tool. A limitation of reaction vectors, however, is that they only account for the structural changes that occur at the reaction centre itself, and they do not consider the presence of more distant functionalities that can affect the transformations. Here, we have combined reaction vector-based de novo design with a Reaction Class Recommender that takes account of whole molecule features of the input molecule to suggest only those reaction class which are more likely to occur in a given environment. The Reaction Class Recommender acts as a filter on the reaction vectors that are applied to a given molecule in de novo design to reduce the number of products that are generated to those which are more likely to be synthesisable in practice. The Reaction Class Recommender has been configured as a multi-label classification problem and trained on starting materials and reaction class labels extracted from reactions in the USPD pharmaceutical patents. A systematic investigation was carried out over a variety of molecular descriptors, label types, multi-label approaches, and classifiers in order to identify the best performing configurations.

The two best performing Reaction Class Recommenders were validated by first applying them to a set of starting materials annotated with their original reaction classes and extracted from the medicinal literature. Both recommenders were shown to be capable of correctly identifying the known reaction classes in just under half the cases. An investigation of the wrong classifications revealed that many of these were also meaningful. No recommendations were made for around 40% of the data with this relatively high percentage likely to be due to the limited coverage of the training data. The high proportion of molecules for which no recommendations was made is not seen as problematic for de novo design, since no filter would be applied on the reaction vectors in this scenario. A simulated de novo design validation was then carried out to demonstrate the effectiveness of the Reaction Class Recommender in an actual design scenario. Significant reductions in the total numbers of structures generated and in the enumeration times were seen while at the same time the estimated synthetic accessibility distributions of the generated structures were shifted toward improved values. Finally, the recommender was used in retrospective de novo design where the aim was to generate known drugs. The target compound was generated in all cases with the recommender resulting in a significant reduction in the total number of compounds generated.

The Reaction Class Recommender has been developed to enhance the de novo design of synthetically accessible molecules and to complement the use of reaction vectors. Whereas the reaction vector focuses on the reaction centre and the structural changes therein, the Reaction Class Recommender is trained on whole molecules and is therefore able to take into account features related to reactivity that are outside of the reaction centre itself. The Reaction Class Recommender has been designed to act as a filter on the reaction vectors that are applied during de novo design and to be used both within a fully automated de novo design cycle and in augmented de novo design.
